# Human papillomavirus proteins are found in peripheral blood and semen Cd20+ and Cd56+ cells during Hpv-16 semen infection

**DOI:** 10.1186/1471-2334-13-593

**Published:** 2013-12-16

**Authors:** Carlo Foresta, Alessandro Bertoldo, Andrea Garolla, Damiano Pizzol, Silvia Mason, Andrea Lenzi, Luca De Toni

**Affiliations:** 1 Department of Molecular Medicine and Centre for Human Reproduction Pathology, University of Padova, Via Gabelli 63, 35121, Padova, Italy; 2 Department of Medical Physiopathology, 1st University of Rome “La Sapienza”, Rome, Italy

**Keywords:** Human papillomavirus, Semen infection, Fluorescence *in situ* hybridization, Peripheral blood mononuclear cells, Hpv-related disease

## Abstract

**Background:**

Human papillomavirus (HPV) currently represents an important risk factor for cancer development and infertility in humans. Whilst binding of HPV to spermatozoa has been associated with male infertility, an investigation about the presence of HPV-DNA in non-spermatozoal semen cells is lacking. Previous findings documented the presence of HPV in peripheral blood leukocytes. The aim of this study was to investigate the expression of HPV markers in semen and blood leukocytes during HPV-16 infection.

**Methods:**

A total of 32 subjects, 16 patients affected by HPV-16 semen infection and 16 controls, were evaluated in our andrological centre and enrolled in the study. Semen non-spermatozoal cells from all subjects were isolated and evaluated for the expression of HPV-16 markers (DNA and L1, E6 proteins) and further characterized for their molecular phenotype. Analogue determination was performed on peripheral blood mononuclear cells.

**Results:**

The presence of HPV-DNA by FISH analysis in a round cell population from semen, confirmed to be CD45+ leukocytes, was observed. These HPV-DNA containing-cells also displayed HPV-16-E6 and HPV-16-L1 viral proteins and, upon further investigation, were found to be CD20+ and CD56+, likely phenotypes of B cells and natural killer cells (NK) respectively. In 25% of the patient group, a very small population of peripheral blood mononuclear cells was found to be positive for HPV-DNA via FISH. These cells displayed the CD20+ and CD56+ phenotype alike. None of the control subjects displayed HPV-DNA in either semen or peripheral blood.

**Conclusion:**

Considering the role of CD20+ and CD56+ cell populations in the antiviral immune response, the detection of HPV markers on leukocytes may reflect the presence of virus particles within the endosomal compartment. However, the presence of HPV markers in circulating mononuclear cells raise concerns about the risk of developing cancers to distal organs.

## Background

Once considered a sexually transmitted virus principally affecting females, human papillomavirus (HPV) currently represents an important factor responsible for both couple infertility and cancer development, as demonstrated by recent experimental and epidemiological data [1]. Cervical cancer is the most common cause of mortality related to human papillomavirus [1] but HPV infection also raises important concerns for men. In fact, HPV has been associated with most male anal cancers (approximately 90%) and a subset of penile (50%) and oral cancers (10–72%) [2]. Additionally, the presence of HPV has been widely reported in semen [3], where it has been associated with impairment of sperm motility [4], supporting the hypothesis that HPV infection is an emerging risk factor for male infertility. Previous studies from our group have documented that HPV can be detected in different cell populations within human semen. By the use of fluorescence *in situ* hybridization (FISH) analysis, HPV-DNA has been detected on both sperm and non-spermatozoal cells [5-7]. These latter include Sertoli cells, squamous epithelial cells and ‘round cells’, such as spermatozoal precursors, erythrocytes and leukocytes [8]. While the presence of Sertoli, immature sperm and epithelial cells is generally considered the result of natural cell turnover and epithelial exfoliation, the presence of increased levels of sperm leukocytes has been traditionally associated with concomitant inflammation or infection of the genito-urinary tract [9]. However, the detection of HPV-DNA in non-spermatozoal cells has not been further investigated. Recent findings from our lab showed that male patients with HPV 16 in semen had a round shaped subpopulation of cells positive for FISH-HPV, not morphologically ascribable to spermatozoa or epithelial cells. A cytological analysis with May Grunwald-Giemsa staining suggested these cells to be of myeloid origin and subsequent cytochemical analysis confirmed the population to be CD45-positive leukocytes. Despite no clear tropism of HPV to white blood cells has been demonstrated, this observation is in agreement with previous reports showing papillomavirus-DNA associated with circulating leukocytes in both human and animal models [10-12]. The possible access of papillomavirus to peripheral blood leukocytes has been very recently strenghtened by experimental data from animal models, showing that BPV-DNA and transcripts can be found in blood cells a few days after intradermal inoculation of horses with BPV1 [13]. Moreover in humans, several studies conducted in female patients with cervical cancers, have shown that HPV DNA can be found in peripheral blood, sera, plasma and arterial cord blood [14,15]. In these studies, authors concluded that HPV detected in blood cells of cancer patients was not, or not-only, subtended by metastatic cells released at a later stage of disease but was also associated to blood mononuclear cells. Moreover, circulating HPV DNA was detected also in healthy blood donors [11]. However, the vast majority of these studies did not show HPV inside blood cells. Most or all of the studies merely focused on the expression of HPV DNA in blood, or on the outside surface of cells [11]. This latter aspect, prompted us to consider HPV infection as a triggering event for virus diffusion into blood stream, even in the absence of evident HPV related diseases. The aim of the present study was to better characterize HPV infected round cells in semen and to evaluate the possible presence of HPV infected cells in blood.

## Methods

### Patients

The study conformed with the principles outlined in the Declaration of Helsinki and was approved by the Institutional Ethics Committee of the Padua General Hospital by Protocol No.2336. All participants provided written informed consent.

Sixteen male patients (mean age 38.8 ± 9.8), attending our Centre for infertility evaluation, were consecutively enrolled in the study on the basis of the detection of HPV-16 in the genito-urinary tract. More precisely, semen samples from patients were formerly assessed for the presence of HPV-DNA on sperm and/or non-spermatozoal cells by FISH analysis. Successively, patients underwent HPV genotyping, performed by INNO LiPA analysis [16], on uretral/coronal sulcus brushing specimens and whole semen samples. Positivity to the sole HPV-16 allowed the enrollment as patients. Exclusion criteria were; history of cryptorchidism, testicular trauma, post-mumps orchitis, HPV-genotype other than HPV-16, presence/history of varicocele or the presence of other urinary/seminal infections detected by testicular Doppler ultrasound and microbiological culture respectively.

Sixteen male partners of infertile couples (mean age 37.5 ± 5.9), negative for any type of HPV infection to genito-urinary as defined above, were recruited as a control group. Peripheral blood samples were collected from every subject evaluated; each sample was assessed for the presence of HPV-DNA as described below.

### Semen sample collection and analysis

Semen samples were obtained by masturbation after 2–5 days of sexual abstinence and stored in sterile containers. Samples were allowed to liquefy for 30 minutes before the evaluation of semen volume, pH, sperm concentration, viability, motility, and morphology according to World Health Organization guidelines for semen analysis [17]. Anti-sperm antibodies were detected using the spermMar test kit for IgG and IgA (FertiPro) as described elsewhere [18].

### Semen processing and round cells isolation

For each subject, isolation of semen round cells was performed by density gradient centrifugation using an Isolate Sperm Separation kit (Irvine Scientific, Santa Ana, CA, USA) according to manufacturer’s instructions. After centrifugation, cells from the upper layer were successively collected, washed in PBS, centrifuged at 300 g for 10 min at room temperature, fixed in 4% paraformaldehyde/PBS solution for 15 min at room temperature, smeared on SuperFrost®Plus glass slides (Menzel Glaser, Braunschweig, Germany), air dried and finally stored at -20°C until further analysis.

### Peripheral blood sampling, processing and peripheral blood mononuclear cell isolation

Peripheral blood samples from HPV16 infected patients and control subjects were drawn from the anterocubital vein in 3 ml Vacutainer-EDTA coated tubes (BD Biosciences, Milano, Italy). For each subject, mononucelar cells from peripheral blood were isolated by density gradient centrifugation on Ficoll Paque Plus (GE Healthcare, Milano, Italy), washed twice in PBS and centrifuged at 300 g for 10 min at room temperature and the pellet was divided in two equal samples. A sample was stored at – 20°C and used for further DNA extraction and PCR analysis. The other sample was fixed in 4% paraformaldehyde/PBS solution for 15 min at room temperature, smeared on SuperFrost®Plus glass slides, air dried and finally stored at -20°C until further analysis.

### Fluorescence in situ hybridization (FISH) for HPV-DNA

Smears of semen round cells and of peripheral blood mononuclear cells, previously fixed in paraformaldehyde were analyzed. To permeabilize cells, samples were digested with pepsin, diluted 1:25,000 in pre-warmed 0.01 mol/L HCl at 37°C. Permeabilization was stopped following 3 to 5 minutes of treatment by rinsing in PBS. Samples were dehydrated in 70% and 100% ethanol for 2 minutes each and finally air dried.

Permeabilized samples were overlaid with 20 μL of hybridization solution containing a biotin-labeled HPV-DNA probe (a mix of total genomes of 7–8 Kb, containing the conserved HPV region, Pan Path, Amsterdam, Netherlands). Each slide was covered with a glass coverslip and the edges were sealed with silicone glue to prevent loss of the mixture during denaturation and hybridization. Following simultaneous denaturation of cellular target DNA and the HPV DNA probe for 5 minutes at 95°C, hybridization was performed by incubating the samples at 37°C overnight in a humidified chamber. Thereafter, the coverslips were carefully removed and the slides were washed in PBS for 10 minutes. The slides were incubated at 37°C for 15 minutes with the differentiation reagent (Pan Path), and washed 3 times in PBS. The biotin-labeled HPV probe was detected by incubation with 1:200 streptavidin Texas Red® (Vector Laboratories, Burlingame, CA) for 40 minutes at room temperature. After detection, the slides were washed twice in PBS/0.01% Triton and then twice in PBS and mounted with a solution containing 5 mg/mL of 4′,6-diamidino-2phenylindole (DAPI) and anti-fade buffer (BioBlue, BioView Ltd., Nes Ziona, Israel). Samples were analyzed using a fluorescence microscope (Nikon ViCo Video Confocal Microscope). For each slide, at least 1000 cells were analyzed. Evaluation of nuclear hybridization signals was performed in triplicate by different investigators. Positive control for HPV-DNA staining, performed on Caski cell line infected with HPV-16, are reported in Additional file 1: Figure S1A.

### PCR amplification of HPV DNA in human mononuclear cells

Isolated mononuclear cells from peripheral blood underwent DNA extraction by the use of a QIAamp DNA mini kit (QIAGEN, Milano, Italy), according to the manufacturer’s instructions. Briefly, sample lysis was obtained following proteinase K digestion at 56°C for 10 min, and the lysates were then loaded on QIAamp columns. After two washes, DNA was eluted with 200 μl of elution buffer. Extracted DNA was kept at -80°C until analysis.

HPV-DNA detection was performed using an Ampliquality HPV-TYPE express kit® (AB Analitica, Padua, Italy) according to the manufacturer’s protocol. The declared detection limit of the assay was ≤ 500 viral copies/reaction and the diagnostic sensitivity was 97,8%.

### Immunofluorescence

Semen round cells and peripheral blood mononuclear cells were analysed as follows. Specimens were treated for 10 min with 1% Triton X-100 (Sigma Aldrich, Milano, Italy) and saturated with 5%BSA-5% Normal Donkey Serum/PBS for 30 min at room temperature. Samples were incubated overnight at 4°C with goat anti-HPV-16 L1 antibody (2 μg/mL), or goat-anti HPV-16 E6 (2 μg/mL) (both from Santa Cruz Biotechnology, Heidelberg, Germany) and/or mouse anti-human CD45, or CD15 or CD4, or CD8, or CD20, or CD56 immunoglobulins (0.4 μg/mL) (all from BD Biosciences). For negative controls, primary antibodies were omitted. Primary immunoreaction was detected following incubation with the appropriate secondary reagent for 60 minutes at room temperature [biotin-conjugated anti-goat antibody and FITC-conjugated anti-mouse antibody, Streptavidin-TexasRed treatment (all 1 μg/mL)]. Samples were finally counterstained with DAPI and mounted in antifade-buffer. Slides were analyzed with a Video-Confocal (VICO) fluorescence microscope (Nikon, Firenze, Italy). Evaluation of nuclear hybridization signals was performed in triplicate by different investigators. Positive controls staining for HPV-E6 and L1 proteins, performed on Caski cell line infected by HPV-16, are reported in Additional file 1: Figure S1A. Positive control staining for human CD45, CD4, CD8, CD20 and CD56, performed on isolated peripheral blood mononuclear rells, are reported in Additional file 1: Figure S1B.

### Statistical analysis

Differences in seminal parameters between patients and controls were evaluated by an unpaired two-sided Student’s *t*-test. Comparisons between frequencies were performed using the χ^2^ test.

Differences were considered significant when P < 0.05. Variables are presented as mean ± SD.

## Results

**Figure 1:**
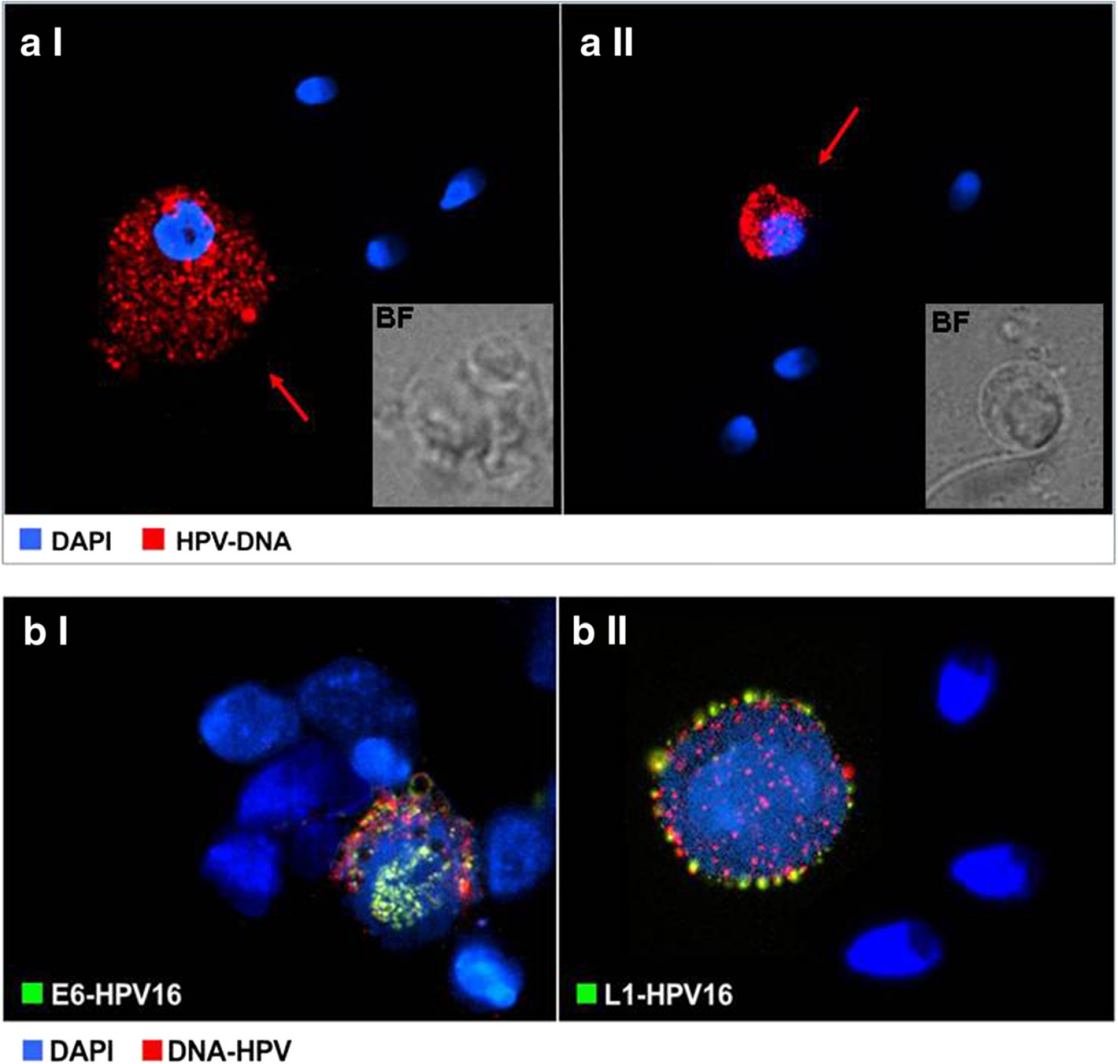
**Analysis of HPV-16 markers in non-spermatozoal cells from semen samples. a**: FISH analysis of HPV-16 DNA in non-spermatozoal cells from semen samples. **a I** epithelial and **a II** non epithelial positive cells. Reference brigh-fiels pictures (BF) are added as inserts. **b:** FISH and immunofluorescence analysis of HPV16-E6 or HPV16-L1 expression on HPV-DNA bearing non-spermatozoal cells. **b I** HPV16-E6 protein and b II HPV16-L1 protein positive round cells.

The seminal parameters of the HPV16-infected patients and control subjects assessed are reported in Table 1. We observed lower sperm motility and an increased frequency of positive SperMar test in HPV16-positive samples. No significant differences in other seminal parameters were observed between the two groups. FISH analysis for HPV, performed on patient semen samples, showed a 26.2 ± 5.9 mean percentage of HPV-DNA bearing sperm. Among non-spermatozoal cells, we observed a mean of 19.3 ± 8.4% HPV bearing cells. The morphological features of these cells were clearly visible by microscopy and were variable, ranging from large-irregular shaped cells with disrupted nuclear margins of likely epithelial origin, to round-regular shaped nucleated cells with limited cytoplasm, possibly of the myeloid lineage (Figure 1a I and II). No infected cells were observed in semen samples from controls.

**Table 1:** Main sperm parameters and mean percentage of HPV infected cells assessed in semen samples from HPV-16 infected patients and control subjects

	**Viability (%)**	**Normal morphology (%)**	**Sperm conc. (10**^**6**^**/ml)**	**Total sperm number (10**^**6 **^**cells)**	**Progressive motility (%)**	**SperMar positive samples (%)**	**Mean% of HPV 16 infected sperm**	**Mean% of HPV 16 infected non-sperm cells**
**HPV-16 positive (n = 16)**	79.2 ± 11.2	19.8 ± 5.8	44.2 ± 15.8	108.9 ± 85.9	28.3 ± 14.3 *	37.5 *	26.2 ± 5.9	19.3 ± 8.4
**Control (n = 16)**	83.1 ± 7.5	19.1 ± 8.0	37.1 ± 9.1	115.1 ± 44.8	40.4 ± 16.8	6.3	0	0

*Abbreviations*: HPV: human papillomavirus.

Significance: * = p < 0.01 vs controls.

### Analysis of semen round cells

No difference between the number of round cells was found comparing the two groups (0.003 ± 0.002 10^3^ cells/ml and 0.002 ± 0.001 10^3^ cells/ml in infected and non infected samples respectively, P > 0.05; Table 2).

**Table 2:** Semen round cells concentration and molecular evaluation of HPV-DNA in semen and peripheral blood mononuclear cells from HPV-16 semen infected and non infected subjects

		**Semen**		**Blood**	
	**Sample number**	**Round cells (x10**^**3**^**/ml)**	**Round cells FISH (+/-)**	**PBMC FISH (+/-)**	**PBMC HPV (+/-)**
**HPV 16 infected samples**	**1**	0,002	+	-	-
	**2**	0,002	+	-	-
	**3**	0,001	+	-	-
	**4**	0,001	+	-	-
	**5**	0,002	+	-	-
	**6**	0,003	+	+	+
	**7**	0,001	+	-	-
	**8**	0,001	+	+	+
	**9**	0,005	+	-	-
	**10**	0,005	+	+	+
	**11**	0,004	+	-	-
	**12**	0,003	+	-	-
	**13**	0,003	+	-	-
	**14**	0,001	+	+	-
	**15**	0,003	+	-	-
	**16**	0,004	+	-	-
	**Average**	**0,003 ± 0,001**			
**Control samples**	**1**	0,001	-	-	-
	**2**	0,002	-	-	-
	**3**	0,005	-	-	-
	**4**	0,001	-	-	-
	**5**	0,002	-	-	-
	**6**	0,004	-	-	-
	**7**	0,003	-	-	-
	**8**	0,001	-	-	-
	**9**	0,001	-	-	-
	**10**	0,003	-	-	-
	**11**	0,001	-	-	-
	**12**	0,000	-	-	-
	**13**	0,003	-	-	-
	**14**	0,003	-	-	-
	**15**	0,002	-	-	-
	**16**	0,001	-	-	-
	**Average**	**0,002 ± 0,001**			

*Abbreviations*: FISH, Fluorescent *in situ* hybridization; HPV, Human Papillomavirus; PBMC, Peripheral Blood Mononuclear Cells; SB:

**Figure 2:**
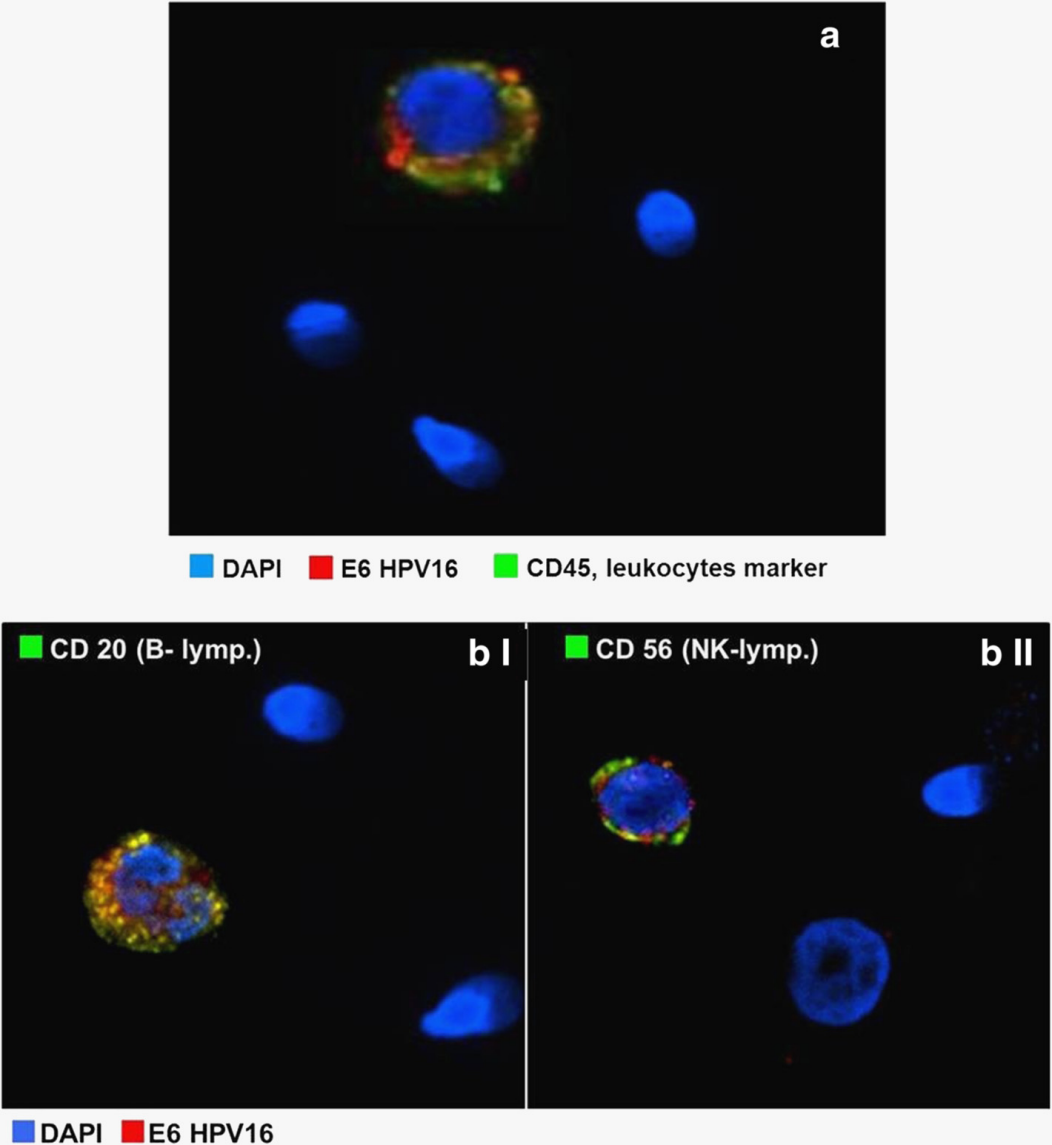
**Immunophenotype of HPV16-bearing cells in semen round cells. a**: Immunofluorescence analysis of HPV16-E6 and CD45 expression on semen round cells. **b**: Immunofluorescence assessment of HPV16-E6 and CD20, or CD56 expression on semen leukocytes.

In order to investigate the molecular phenotype of HPV bearing cells, we enriched samples in round cells by performing a density gradient centrifugation of whole semen. The recovered number of semen round cells was not quantitative and varied greatly within the cohort of infected and non infected subjects. FISH analysis performed on round cells detected HPV-DNA in all HPV 16 infected samples; no specific signal was observed in controls. The number of round cells displaying HPV-DNA varied from 10 to 40% among isolated cells. HPV-DNA was located principally in the cytoplasm and less frequently in or over the nucleus (Figure 1b I-II). Moreover, in almost all HPV-DNA positive round cells, a signal for both E6- and L1-HPV16 proteins was detected. Similar to HPV-DNA, viral proteins were mainly located in the cytoplasm and less frequently in the nucleus. An immunofluorescence assay for CD45 and E6 expression performed on the same specimens (Figure 2a) demonstrated that the vast majority (>95%) of E6-positive round cells were CD45-positive leukocites [19]. Additional immunofluorescence assays (Figure 2b I-II) on HPV-positive mononuclear cells expressing the E6 protein, rarely or never displayed staining for CD4 or CD8 cell markers. In contrast, specific staining for CD20 or CD56 markers were found on 20% and 70% of E6 positive cells respectively. Moreover, nearly the 80% of semen CD56+/CD20+ leukocytes displayed HPV16-E6 protein.

### Analysis of peripheral blood leukocytes

**Figure 3:**
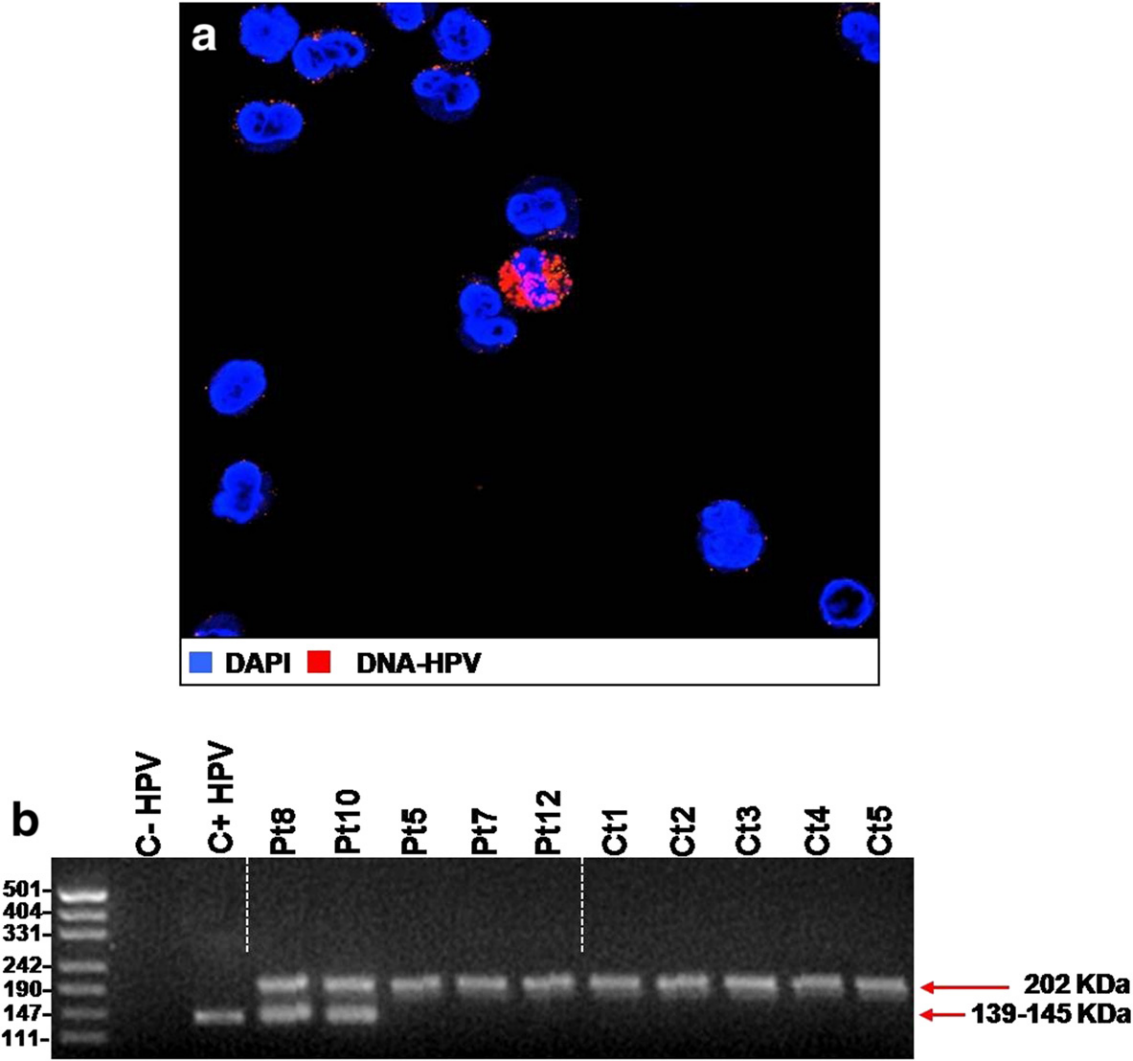
**Analysis of HPV-DNA in peripheral blood mononuclear cells. a**: FISH analysis of HPV-DNA in peripheral blood mononuclear cells. **b**: PCR assessment of HPV-DNA in peripheral blood mononuclear cells from patients and controls. An example of HPV-DNA product (139–145 kDa) detectable in patients 8 and 10 (Pt8 and Pt10 respectively) and undetectable in patients 5, 7 and 12 as well as in all control subjects (Pt5, Pt7, Pt12, Ct1-5 respectively). Thiosulfate sulfurtransferase (TST) band at 202 kDa was used as an internal control.

**Figure 4:**
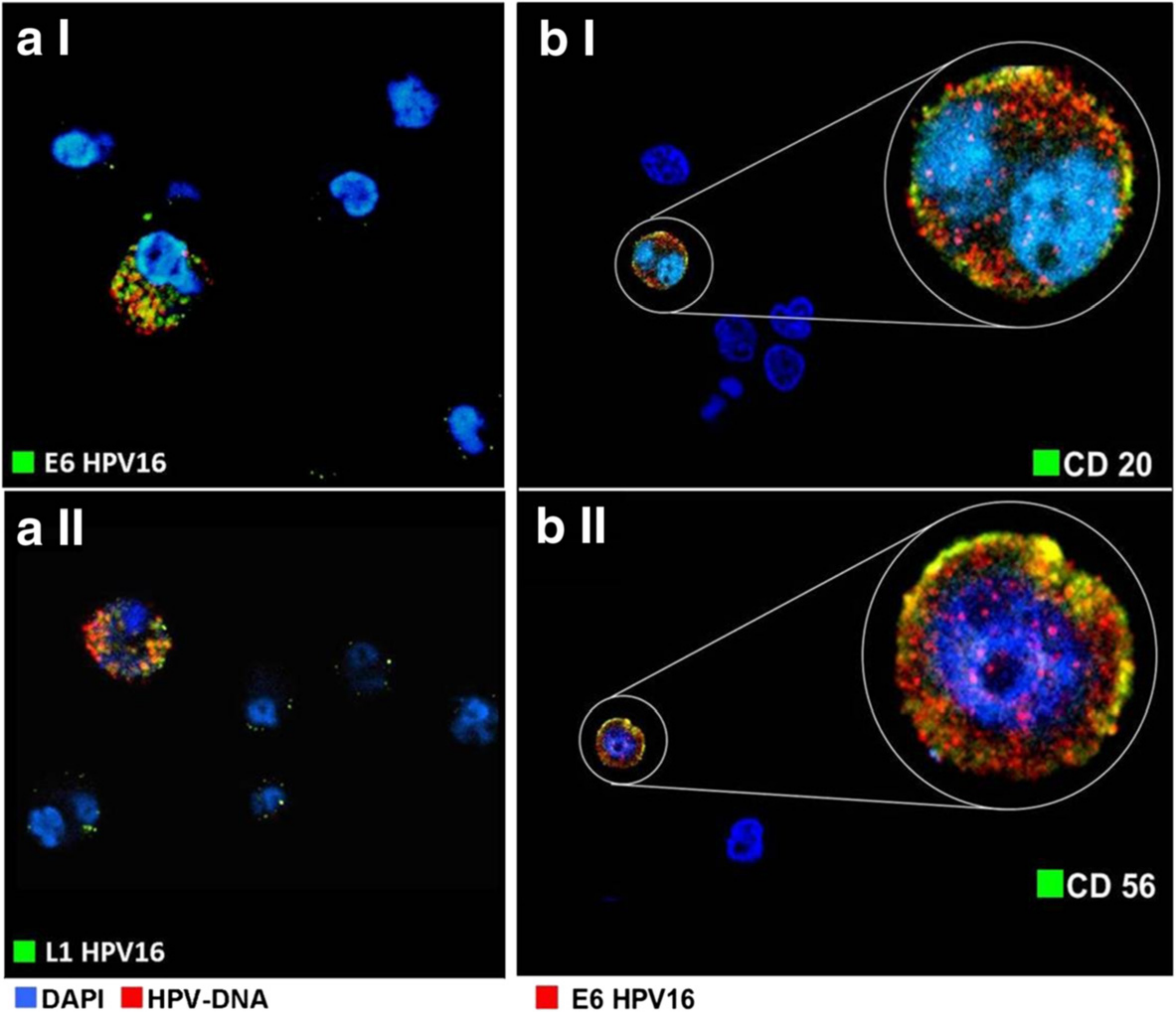
**Immunophenotype of HPV16-bearing cells in peripheral blood mononuclear cells. a**: Immunofluorescence for HPV16-E6 and HPV16-L1 proteins on peripheral blood mononuclear cells. **a I** HPV16-E6 and **a II** HPV16-L1 positive mononuclear cells. **b**: Immunofluorescence for HPV16-E6 and CD20 or CD56 on peripheral blood mononuclear cells. **b I** HPV16-E6 and CD20 positive mononuclear cells. **b II** HPV16-E6 and CD56 positive mononuclear cells.

The presence of HPV-DNA in peripheral blood mononuclear cells (PBMC) was initially assessed by FISH. As reported in Table 2, 4 patients out of 16 (25%) featured by HPV-16 semen infection showed specific staining for HPV-DNA in a very restricted population of PBMC ranging from 0.1-0.6% (Figure 3a). In control subjects, the presence of HPV-DNA in PBMC was never detected. In parallel, the presence of HPV-DNA in peripheral blood mononuclear cells was evaluated by PCR. Single-step PCR analysis showed the presence of HPV-DNA in 3 out 4 PBMC samples showing HPV-DNA detected by FISH (Figure 3b) None of controls showed positive reaction. Further immunofluorescence analysis demonstrated that HPV-DNA localised in the cytoplasm of positive cells and co-localized with E6- and L1-HPV16 proteins in almost all HPV-DNA-bearing cells (Figure 4a I-II). Finally, the molecular phenotype of PBMC found to express HPV-E6 protein was investigated by immunofluorescence. E6-positive cells were found to co-express CD20 or CD56 in 35% and 60% of cases respectively (Figure 4b I-II). CD8 was found to be expressed by 5% of E6 positive cells while in none of the samples was found the co-expression of CD4 and E6 (data not shown).

## Discussion

The results of this study have identified, for the first time, the presence of HPV16 in CD45+ leucocytes in the semen of infected patients. Furthermore, HPV was also detected in a very restricted subpopulation of circulating PBMC bearing a similar immunophenotype. Although these findings are in contrast with the known tropism of HPV for epithelial cells [20], other authors have reported HPV spreading both from cancers and infected sites [10,20]. Pao et al. [21], first reported the presence of different types of HPV [6,11,16,19] in peripheral blood mononuclear cells from female patients affected by HPV infections of the genito-urinary tract [21]. In addition, in a recent study performed on HIV patients, 11 of 57 blood samples were positive for HPV-16 DNA [14]. The authors stated that the HPV genome detected in positive samples existed as an episomal form, albeit at a low DNA copy number. Moreover, in another study assessing a cohort of healthy Australian blood donors, the presence of HPV-DNA in peripheral leukocytes was reported in approximately 8% of subjects [11]. Presence of circulating HPV can be explained by the immune response. Local and systemic viral infections are controlled by innate and adaptive immune mechanisms, where both humoral responses, which prevent disease recurrence [22], and cell-mediated immune response are triggered. In particular for cell-mediated immunity, NK cells together with CD1d-restricted NK-T cells (iNKT cells) are the main cell populations of the innate immune system responsible for the clearance of HPV infection [23]. NK cells constitutively express IFNγ, granzyme and perforin. They are ‘primed’ to initiate an anti-viral immune response by detecting decreased MHC-I expression on infected cells, in the case of HPV, due to repression by the E5 and E7 viral-oncoproteins at specific sites of the MHC-I processing and presentation pathway [22-25]. In parallel, the activation of antigen presenting cells (APC) and subsequent antigen presentation in the draining lymph nodes is necessary for the initiation of a primary adaptive response to an infecting agent. In the case of HPV, the local APCs responsible are the Langherhans cells of the epidermis, dendritic cells present in the dermis, and occasionally CD20+ B-lymphocytes [25-28]. Data from animal models of papillomavirus infection suggest that APCs take up virus-like-particles through the FcγRIII receptor [29], degrade antigen within proteasomes and incorporate it into MHC II on the cell surface. Thereafter, APCs trigger the T cell-mediated adaptive response of CD4+ and CD8+ populations [29,30], in part by the release of a typical pattern of inflammatory cytokines such as interleukin-1β, interleukin-12, tumour necrosis factor-α, and interleukin-6 [31]. In this study, the presence of human papillomavirus markers in a subset of semen leukocytes from a cohort of HPV-genitourinary infected patients has been documented. Both HPV-16 E6 and L1 proteins co-localized with the viral DNA. Notably, cells displaying molecular hallmarks of HPV were frequently found to be CD20+ B-lymphocytes and CD56+ natural killer-like (NK-like) cells, two leukocyte populations recruited during viral infection. In addition, in peripheral blood of 25% of patients, the presence of HPV-bearing cells was found by FISH and further confirmed by single-step PCR. In agreement with the results observed in semen, DNA and protein markers of HPV in peripheral blood were mainly associated with CD20+ and CD56+ cells. It is possible that signals for viral DNA and L1-protein could be likely due to the endocytic uptake of virions, given that they are mostly observed as punctate patterns in the cytoplasm. However, if this was the case, virion endocytosis does not explain the detection of E6 protein and it is highly unlikely that the observed E6 signal is an artefact since it is only seen in HPV DNA positive cells. These evidences raise the hypothesis that NK-like cells and B lymphocytes represent possible targets of HPV, in according to previous studies [32,33]. In fact on a side, Renoux et al. [32] showed by *in vitro* studies that cytotoxic activity and cytokine production by NK cells appeared to be linked to rapid HPV-VLP entry into these cells by macropinocytosis. Since CD16 blockade inhibited this process, the authors concluded that CD16 is necessary for HPV–VLP internalization [32], even if a role for CD16 as a primary receptor for HPV infection has not yet been demonstrated. On the other side, the competency of HPV to be uptaken by B lymphocytes cannot be excluded. In fact, this possibility is supported by the fact that heparan-sulfate proteoglycans, theorised to potentially be one of the primary receptors involved in HPV entry, are expressed by B lymphocytes and are a requirement for normal cell maturation, differentiation and function [33]. However, recent studies suggested that HPV entry into target cells is actually the result of a concerted interaction between virus-capside proteins and more than one receptor on cell surface like α6-integrin or annexin A2-heterotetramer [34,35]. Thus further investigation will be necessary to clarify this aspect.

It has been postulated that HPV in polymorphonuclear cells may be involved in vertical transmission of the virus, following the detection of HPV-DNA in reproductive and placental cells, as well as in virgins, infants and children [36,37]. Indeed, since HPV-DNA has been detected in amniotic fluid [37], placenta and the umbilical cord [36], the hematogenous route of chorionic and placental tissue infection and subsequent spread to amniotic cells and possible ingestion by the fetus has been hypothesized [36,37]. Since sporadic fetal brain malformations (eg, dysplasias) could be detected as early as 24 weeks gestation [38], it has been suggested the transplacental spread of HPV16 as a plausible mechanism for entry into the brain during early fetal cortical development, even in the absence of overt clinical infection. Interestingly, HPV-16 protein expression has very recently been reported in patients affected by focal cortical dysplasia type IIB (FCDIIB) [39]. In particular, HPV16-E6 protein was robustly expressed in cerebral cortex specimens of all FCDIIB patients examined, most notably in the cytoplasm of balloon cells (BC). E6 protein signals were not detectable in regions without BC or in control tissue specimens. Moreover, E6 expression in BC displayed a strong association with components of the mammalian target of rapamycin complex 1-signalling pathway, whose abnormal activation is linked to FCDIIB etiology [40]. Taken together, these observations pose an open question regarding the meaning of HPV markers found in distinct blood leukocyte populations as the result of cell-mediated immunity or the involvement in virus spreading from the original site of infection.

In addition to the low number of patients enrolled, the main drawback of this study is represented by the extremely low percentage of HPV positive leukocytes recovered in seminal fluid and peripheral blood. A possible reason for these findings is that semen immune cells can become unproductively infected with HPV16 when trafficking through the seminal compartment in HPV16-infected men, and these cells may traffic back into circulation and be detected at low frequency in peripheral blood. This condition limited our ability to isolate HPV bearing leukocytes and further evaluate their infectiveness. Thus a mechanistic explanation about how virus enters circulating mononuclear cells remains an open question. Moreover, the assessment of HPV DNA on peripheral blood performed by INNO LiPA was not able to confirm the results obtained by FISH and Single-step PCR analysis (data not shown). A possible explanation of this discrepancy may reside in the different method sensitiveness.

## Conclusion

In conclusion, this is the first study reporting the presence of HPV-antigens together with HPV-DNA in circulating PBMC from male patients with HPV-16 infection of the genitourinary-tract. This evidence raises major concerns about the risk of developing cancers to distal organs.

## Competing interests

The authors declare that they have no competing interest.

## Authors’ contributions

CF supervised the study; DP performed outpatients evaluation; AB performed laboratory evaluation; SM carried out the molecular genetic studies; AL supervised manuscript editing; CF, LDT, AG, DP drafted the manuscript. All authors read and approved the final manuscript.

## Pre-publication history

The pre-publication history for this paper can be accessed here:

http://www.biomedcentral.com/1471-2334/13/593/prepub

## Supplementary Material

Additional file 1: Figure S1 **A:** Positive FISH staining for HPV-DNA and immunofluorescence staining for HPV-16 E6 an L1 proteins in cultured Caski cells infected with HPV 16. **B:** Positive immunofluorescence staining for human CD45, CD20, CD4, CD8 and CD56 antigens performed in isolated peripheral blood mononuclear cells. Click here for file
