# Supplementary figures and images for: Human papillomavirus proteins are found in peripheral blood and semen Cd20+ and Cd56+ cells during Hpv-16 semen infection
Source: BMC Infect Dis. 2013 Dec 16;13:593. doi: 10.1186/1471-2334-13-593 (PMC3878630; doi:10.1186/1471-2334-13-593)

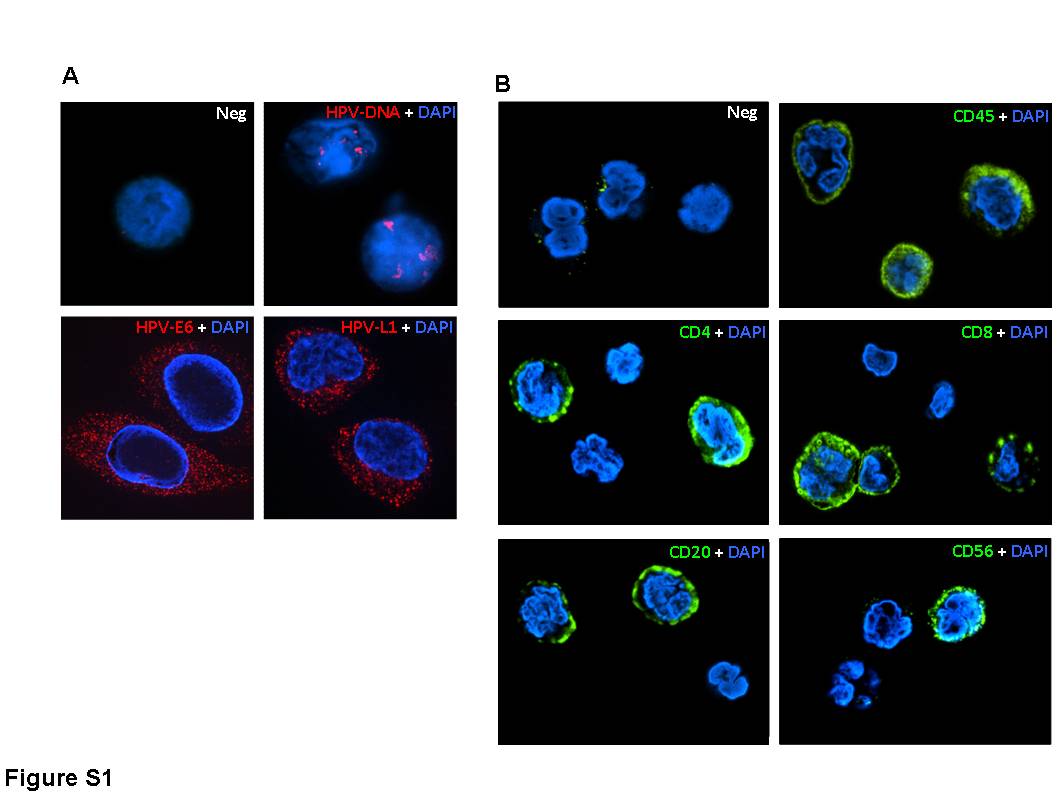

Supplement: Additional file 1: Figure S1 — A: Positive FISH staining for HPV-DNA and immunofluorescence staining for HPV-16 E6 an L1 proteins in cultured Caski cells infected with HPV 16. B: Positive immunofluorescence staining for human CD45, CD20, CD4, CD8 and CD56 antigens performed in isolated peripheral blood mononuclear cells. [file 1471-2334-13-593-S1.jpeg]
